# Using Electrical Impedance Myography as a Biomarker of Muscle Deconditioning in Rats Exposed to Micro- and Partial-Gravity Analogs

**DOI:** 10.3389/fphys.2020.557796

**Published:** 2020-09-15

**Authors:** Carson Semple, Daniela Riveros, Dong-Min Sung, Janice A. Nagy, Seward B. Rutkove, Marie Mortreux

**Affiliations:** Department of Neurology, Harvard Medical School – Beth Israel Deaconess Medical Center, Boston, MA, United States

**Keywords:** EIM, muscle, impedance, rats, spaceflight, ground-based, hindlimb unloading, partial weight-bearing

## Abstract

As astronauts prepare to undertake new extra-terrestrial missions, innovative diagnostic tools are needed to better assess muscle deconditioning during periods of weightlessness and partial gravity. Electrical impedance myography (EIM) has been used to detect muscle deconditioning in rodents exposed to microgravity during spaceflight or using the standard ground-based model of hindlimb unloading via tail suspension (HU). Here, we used EIM to assess muscle changes in animals exposed to two new models: hindlimb suspension using a pelvic harness (HLS) and a partial weight-bearing (PWB) model that mimics partial gravity (including Lunar and Martian gravities). We also used a simple needle array electrode in lieu of surface or *ex vivo* EIM approaches previously employed. Our HLS results confirmed earlier findings obtained after spaceflight and tail suspension. Indeed, one EIM measure (i.e., phase-slope) that was previously reported as highly sensitive, was significantly decreased after HLS (day 0: 14.60 ± 0.97, day 7: 11.03 ± 0.81, and day 14: 10.13 ± 0.55 | Deg/MHz|, *p* < 0.0001), and was associated with a significant decrease in muscle grip force. Although EIM parameters such as 50 kHz phase, reactance, and resistance remained variable over 14 days in PWB animals, we identified major PWB-dependent effects at 7 days. Moreover, the data at both 7 and 14 days correlated to previously observed changes in rear paw grip force using the same PWB model. In conclusion, our data suggest that EIM has the potential to serve as biomarker of muscle deconditioning during exposure to both micro- and partial- gravity during future human space exploration.

## Introduction

It has long been known that the muscular system is profoundly impacted by weightlessness (Edgerton et al., 1995; Baldwin, 1996), resulting in rapid and significant atrophy (LeBlanc et al., 1995; Fitts et al., 2001) that is especially pronounced in the weight-bearing triceps surae muscles (i.e., gastrocnemius and soleus) (Desplanches et al., 1990; Desplanches, 1997). Several ground-based models in humans (e.g., head-down bed rest, HDBR) (Bloomfield, 1997; Ploutz-Snyder et al., 2014; Hargens and Vico, 2016; Ploutz-Snyder, 2016) and rodents (e.g., hindlimb unloading via tail suspension, HU) (Kasper, 1995; Chowdhury et al., 2013; Globus and Morey-Holton, 2016) have allowed scientists to unravel the cellular mechanisms at play (Han et al., 2007; Giger et al., 2009; Momken et al., 2011; Hanson et al., 2013). Numerous countermeasures have been investigated and refined in such analog models, including exercise (Swift et al., 2010; Fujita et al., 2011), nutraceuticals (Momken et al., 2011), pharmacological agents (Tipton and Sebastian, 1997), or a combination of several of these approaches (Li et al., 2012; Dillon et al., 2018), in order to lessen muscle loss and dysfunction during periods of microgravity. These findings led to the establishment of strict and tailored exercise regimens for astronauts (Trappe et al., 2009; Petersen et al., 2016, 2017; Lambrecht et al., 2017; Lang et al., 2017), rendered possible by the exhaustive equipment available for all crewmembers onboard the ISS (Korth and Reeves, 2015). Indeed, astronauts typically exercise 2 h/day in-flight, using the ARED (Advanced Resistive Exercise Device), T2 (T2 Combined Operational Load Bearing External Resistance Treadmill), and CEVIS (Cycle Ergometer with Vibration Isolation System) devices, allowing preservation of muscle mass and function during their missions. These exercises, in combination with pre-flight conditioning and post-flight rehabilitation, also help ensure muscle function upon return to terrestrial gravity.

Space agencies are now preparing for more extensive extra-orbital missions. NASA, with the Artemis program, plans to send the first woman and next man on the Lunar surface in 2024, build the Gateway orbital station, and eventually launch the first manned-Mars exploration in the 2030 s (National Aeronautics and Space Administration, 2007). The Moon and Mars have partial gravity (0.16 and 0.38 *g*, respectively), and will present new challenges for astronauts, accustomed to either 0 or 1 *g*. Two ground-based models of partial weight-bearing (PWB) have been developed to investigate the effects of partial mechanical loading on the muscular system, one in mice (Ellman et al., 2013) and one in rats (Mortreux et al., 2018, 2019a,c; Semple et al., 2020). These studies have shown a near linear effect of PWB level (Mortreux et al., 2018, 2019a) on muscle loss, without revealing the existence of a threshold, however, some mitigating strategies display encouraging results (Mortreux et al., 2019b).

Clearly there is a need for small, non-invasive diagnostic tools that will allow monitoring of muscle health and condition during all upcoming missions. Electrical impedance myography (EIM) is a technology that has been used in both pre-clinical (Li et al., 2016; Kapur et al., 2018b; Nagy et al., 2018; Clark-Matott et al., 2019; Mortreux et al., 2019d) and clinical studies (Spieker et al., 2013; Mcilduff et al., 2017; Rutkove et al., 2017; Sanchez and Rutkove, 2017a; Shefner et al., 2018). By applying a high-frequency, low-amplitude electrical current in the muscle and recording the resulting voltages, muscle quality can be assessed non-invasively (Sanchez and Rutkove, 2017b). EIM has been used previously in mice returning from a 13.5-day orbital trip (Sung et al., 2013) and in rats with tailed-based HU (Li et al., 2013). In both cases, EIM was able to detect comparable changes associated with muscle atrophy and weightlessness-induced disuse. Here, we used a novel, easily-applied needle EIM array to longitudinally assess the gastrocnemius muscle of rats in two new relevant models of mechanical unloading: (1) hindlimb suspension via pelvic harness (HLS) rather than the well-established tail-suspension (HU) approach; and (2) exposure to different levels of PWB for 14 days using a recently developed suspension apparatus. Our hypotheses were: 1. EIM analysis using a needle array will replicate earlier findings previously obtained in space-flown and HU rodents and 2. PWB will generate dose-dependent effects on EIM values. Proof of these two hypotheses would provide additional support for the potential use of EIM in humans to assess muscle health during long-duration space flight as well as Lunar or Martian exploration.

## Materials and Methods

### Animals

All experimental protocols were approved by the Beth Israel Deaconess Medical Center Institutional Animal Care and Use Committee under the authorization numbers 067-2016 and 025-2019. 13-week-old male Wistar rats (Charles River Laboratories, Wilmington, MA, United States) were obtained and housed in a temperature-controlled room (22 ± 2°C) with a 12:12 h light-dark cycle starting at 7:00 am and housed in the facility for 1 week prior to the experiments. Water and chow were provided *ad libitum* and monitored daily. Rats were individualized and allowed to acclimate to their custom-cage and apparatus for 48 h before being exposed to different levels of partial weight-bearing (PWB) or to hindlimb suspension (HLS).

For all experiments requiring anesthesia, inhaled isoflurane (1.5–3.5%) + oxygen was used, while body temperature was maintained constant using a water-controlled therapy pad bath (Gaymar, Orchard Park, NY) set at 37°C (Fisher Scientific, Hampton, NH, United States). Before EIM measurements, rats were placed in a prone position with the left hind leg taped at a 45° angle from the spine and the fur overlaying the skin was clipped. At the end of the experiment, rats were euthanized by CO_2_ inhalation according to IACUC guidelines.

### Partial Weight-Bearing (PWB) and Hindlimb Suspension (HLS)

On day 0 (baseline), forty (40) rats were divided into 4 different loading groups: normal loading (PWB100, terrestrial gravity), 40% of normal loading (PWB40, Martian gravity analog), 20% of normal loading (PWB20, Lunar Gravity analog), and 70% of terrestrial gravity (PWB70, an intermediate between Martian and terrestrial gravity values) and followed for 14 days. A detailed description of the housing environment and suspension apparatus can be found in our article describing this model (Mortreux et al., 2018). Briefly, rats of all groups are housed in single cages with a floor surface of 12”×12” and 16” high. Groups were composed of 8 animals each (*n* = 16 for PWB100) and allocated to ensure equal distribution of the body weights. Achieved PWB level was calculated daily for each animal by obtaining its fully-loaded weight and its unloaded weight successively using a digital animal scale and then calculating the ratio between unloaded and fully-loaded values, as described elsewhere (Mortreux et al., 2019a). Deviations greater than 5% of the desired PWB level were corrected by adjustment of the chain length in the suspension apparatus. An additional twenty-eight (28) rats were placed in hindlimb suspension (HLS) using a pelvic harness as previously described (Mortreux et al., 2019c). Throughout the manuscript, HU refers to tail-suspension while HLS refers to hindlimb suspension using a pelvic harness.

### Rear Paw Grip Force (RPGF)

Functional testing was obtained separately on 149 outbred Wistar male rats (14-week-old) exposed to various degrees of PWB, and used to determine the longitudinal time-course of muscle deconditioning (Mortreux et al., 2019a). Weekly, rear paw grip force was assessed with a 50 N capacity digital force meter (Chatillon, Largo, FL, United States). Animals were positioned so that their rear paws grip the force transducer are gently pulled backwards until they release their grip and the peak force was recorded. Three consecutive trials were performed with a short latency period, and the results of the 3 trials were averaged and compared to baseline (day 0, pre-suspension) values.

### Electrical Impedance Myography (EIM)

*In vivo* impedance measurements were performed weekly under 1.5% anesthesia using the mView System (Myolex, Inc., Boston, MA, United States), and obtained at 41 frequencies ranging from 1 kHz to 10 MHz. The output includes the values of phase (LP), reactance (LX) and resistance (LR) for each frequency. Measurements were made using a fixed 4 mm wide needle array (3 mm deep, 1 mm of the distal tips left exposed) that was inserted along the length of the left gastrocnemius muscle. The array was assembled from a series of subdermal 27G needle electrodes (Ambu, Neuroline, Copenhagen, Denmark) and the needles were coated evenly with a non-conductive lacquer leaving only the tip exposed, thus reducing the impact of the subcutaneous fat. 50 kHz EIM values were analyzed including phase (LP), reactance (LX), and resistance (LR). Multifrequency analysis was performed and slopes were calculated from the EIM values obtained between 100 and 500 kHz. AUC LX was calculated based on reactance vales for frequencies from 5000 to 500,000 Hz.

### Statistical Analyses

All data were analyzed using GraphPad Prism 8.4 (GraphPad Software, La Jolla, CA, United States) using one-way or two-way repeated measures (RM)ANOVA (or mixed models) and followed by *post hoc* tests (Tukey’s and test for linear trend), and considered significant when *p* < 0.05. Correlations were performed based on average values, and Spearman coefficients were calculated.

## Results

### Hindlimb Suspension Alters EIM in Parallel With Grip Force Reductions

**FIGURE 1:**
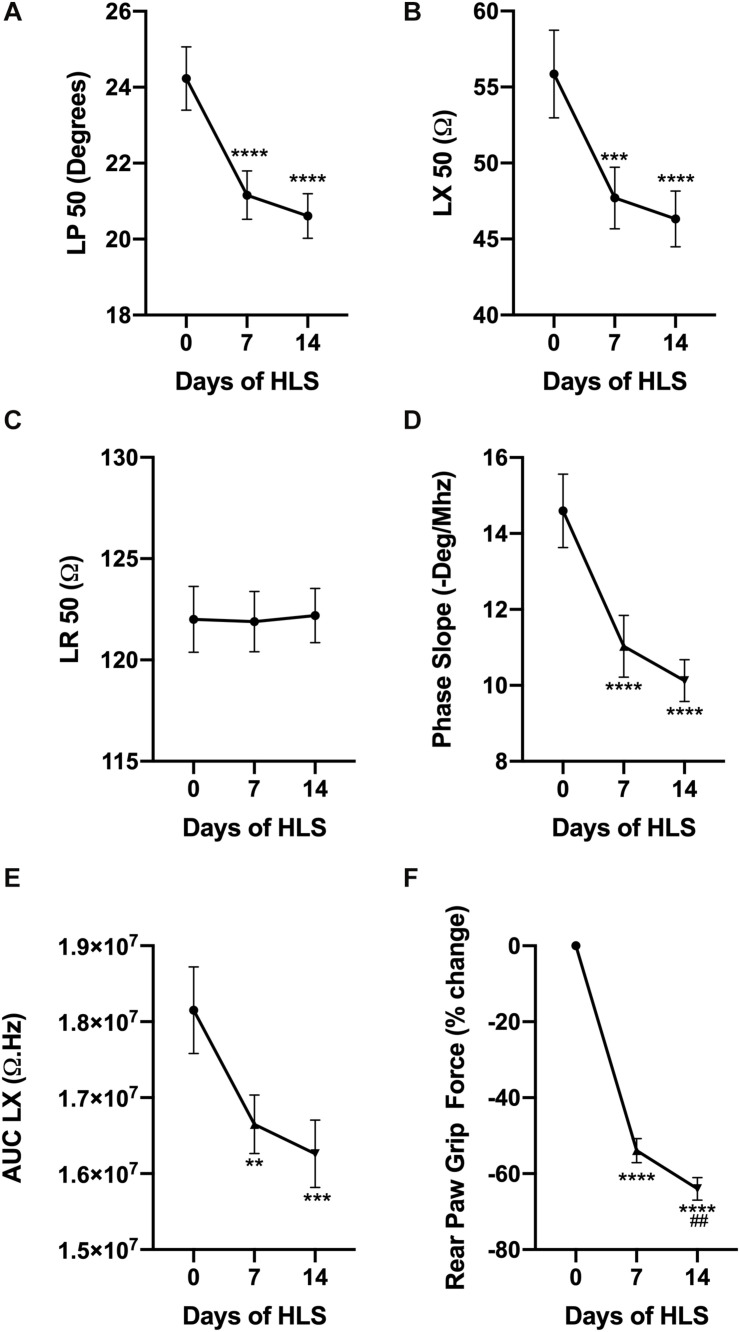
HLS induces a decrease in muscle force and alters EIM values and parameters. Evolution of EIM values at 50 kHz during 14 days of exposure to HLS including longitudinal phase (LP, **A**), longitudinal reactance (LX, **B**), and longitudinal resistance (LR, **C**). Calculated parameters based on multifrequency analysis including phase-slope (P-slope, **D**) and area under curve of the reactance (LX AUC, **E**). Change in rear paw grip force (RPGF) during 14 days of exposure to HLS **(F)**. All results are displayed as mean ± SEM with *n* = 28. Data were analyzed using a 1-way repeated measures ANOVA and followed by Tukey’s *post hoc* tests. Results of Tukey’s tests are displayed on the graph as **, ***, ****p < 0.01, *p* < 0.001, *p* < 0.0001 vs. day 0, respectively and as ^##^*p* < 0.01 vs. day 7.

Fifty kilohertz EIM values were measured weekly including longitudinal phase (LP, Figure 1A), longitudinal reactance (LX, Figure 1B), and longitudinal resistance (LR, Figure 1C) to assess the effects of muscle disuse. While LP and LX displayed a sharp and significant decrease after 7 and 14 days of unloading, LR remained steady throughout the HLS experiment. Similarly, two parameters that are calculated from the EIM data, i.e., the phase-slope (Figure 1D) and the area under the curve of the longitudinal reactance (AUC LX, Figure 1E) significantly decrease during unloading. These changes are consistent with those observed previously in our HLS experiments and are associated with a rapid loss in RPGF (Figure 1F). Indeed, grip force declines by 54% after only 7 days of suspension, and progressively worsens to reach a 64% reduction from baseline after 14 days of HLS.

### Partial Weight-Bearing Alters EIM Parameters in Subtler and More Complex Ways, Also in Parallel With Grip Force Changes

**FIGURE 2:**
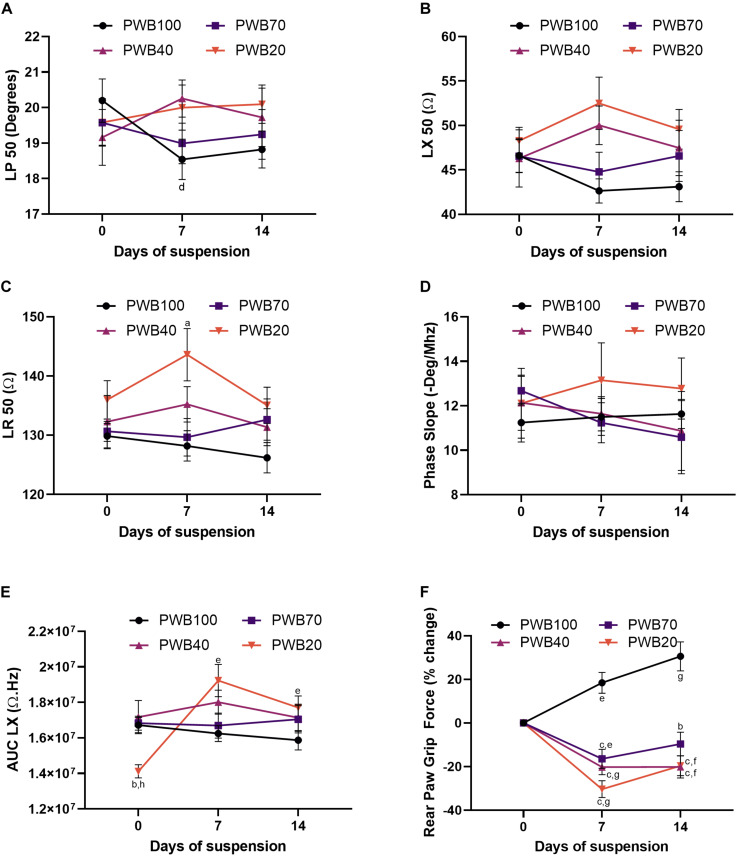
PWB induces a decrease in muscle force which is not seen in traditional EIM values and parameters. Evolution of EIM values at 50 kHz during 14 days of exposure to HLS including longitudinal phase (LP, **A**), longitudinal reactance (LX, **B**) and longitudinal resistance (LR, **C**), *n* = 7–16 per group. Calculated parameters based on multifrequency analysis including phase-slope (P-slope, **D**) and area under curve of the reactance (LX AUC, **E**), *n* = 7–16 per group. Change in rear paw grip force (RPGF) during 14 days of exposure to PWB **(F)**, *n* = 24–39 per group. All results are displayed as mean ± SEM. Data were analyzed using a 2-way repeated measures ANOVA and followed by Tukey’s *post hoc* tests. Results of Tukey’s tests are displayed on the graph as a, b, c: *p* < 0.05, *p* < 0.001, *p* < 0.0001 vs. PWB100, respectively; d, e, f, g: *p* < 0.05, *p* < 0.01, *p* < 0.001, *p* < 0.0001 vs. day 0, respectively; and h: *p* < 0.001 vs. PWB70. Details of the ANOVA analysis can be found in the Supplementary Table 2.

In animals exposed to PWB, EIM values for phase, reactance and resistance at 50 kHz did not significantly vary over the 14-day period of suspension (Figures 2A–C, however, small but significant changes were detected at 7 days (discussed below). Unlike in animals exposed to HLS, phase-slopes were not significantly altered by exposure to PWB (Figure 2D); and the area under curve of the longitudinal reactance (AUC LX, Figure 2E) remained steady over 2 weeks of partial weight bearing in the PWB100, PWB70, and PWB40 groups. However, significant variations in AUC LX were observed in the PWB20 group throughout the experiment, without establishing a clear direction over time. Despite this variability, animals displayed a significant loss in RPGF at 7 days at every PWB level, apart from the control group that displayed a constant increase from baseline over the 2-week period of observation (Figure 2F). However, unlike the HLS animals, rats exposed to PWB generally improved their hindlimb grip force during the second week of unloading (Figure 2F).

**FIGURE 3:**
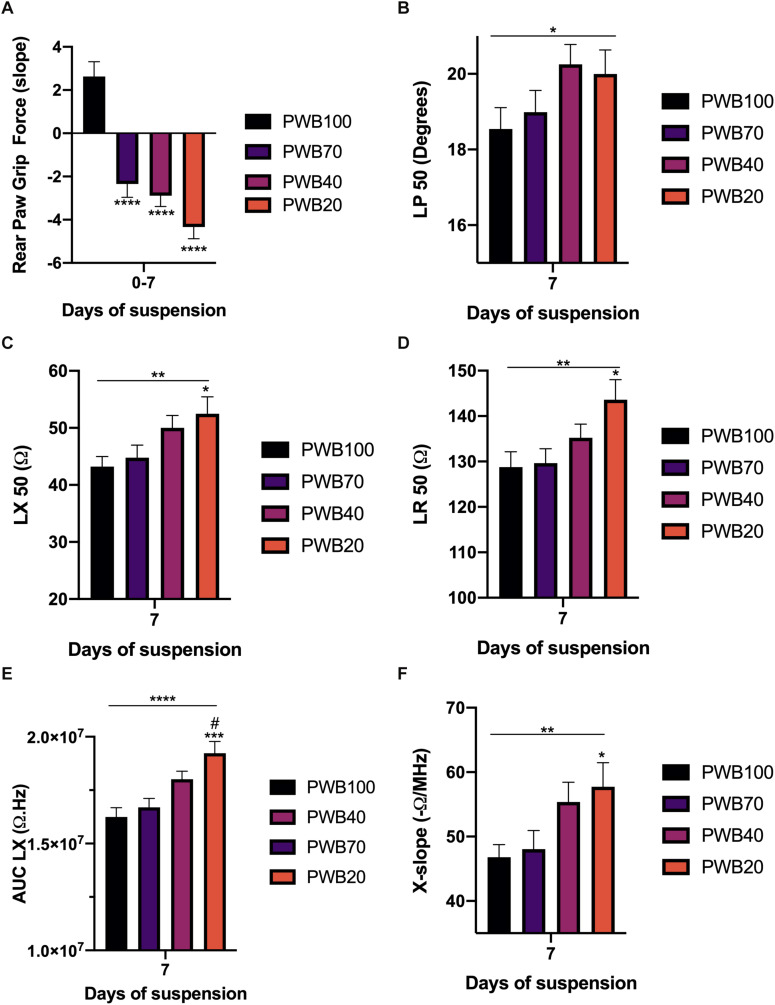
The strongest muscle deconditioning occurs during the first week of PWB and is associated with significant alterations in EIM values and parameters. Slope for the change in rear paw grip force during the first week of exposure to PWB **(A)**. 50 kHz EIM values at 7 days of exposure to PWB including longitudinal phase (LP, **B**), longitudinal reactance (LX, **C**), and longitudinal resistance (LR, **D**). Calculated parameters at 7 days of exposure to PWB including area under curve of the reactance (LX AUC, **E**) and reactance-slope (X-slope, **F**). All results are displayed as mean ± SEM with *n* = 7–16 per group except for panel A (*n* = 35–39 per group). Data were analyzed using a 2-way repeated measures ANOVA and followed by Tukey’s *post hoc* tests. Results of Tukey’s tests are displayed on the graph as *, **, ***, ****: *p* < 0.05, *p* < 0.01, *p* < 0.001, *p* < 0.0001 vs. PWB 100, respectively; #: *p* < 0.05 vs. PWB70. Lines represent the results of the linear *post hoc* test across all groups. Detailed statistics of the ANOVA can be found in Supplementary Table 2.

As the detrimental effects of PWB, based on RPGF, occurred mainly during the first week of exposure and since our animals all displayed relatively similar EIM values at baseline (Supplementary Table 1), we next focused our analysis on 7-day EIM values. A clear dose-dependent linear increase in LP (Figure 3B), LX (Figure 3C), and LR (Figure 3D) at 50 kHz was present. Critically, all of these changes were in the *opposite* direction compared to animals exposed to HLS, with the PWB20 showing the greatest change. Similarly, this dose-dependent increase was found in our multifrequency analysis as shown in the AUC LX (Figure 3E). Unlike animals exposed to HLS, phase-slopes did not appear sensitive to muscle disuse during the first week of PWB, however, reactance-slope, a different multifrequency measure previously used to assess muscle health (Li et al., 2013) (X-slope, Figure 3F) displayed a linear relation to decreasing PWB levels.

**FIGURE 4:**
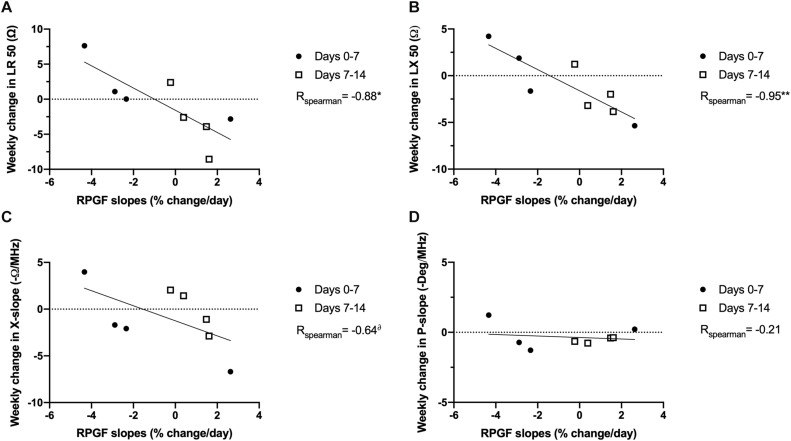
EIM variability is correlated to functional changes in grip force in rats exposed to PWB. Correlations between evolution of EIM parameters and the slope of the change in rear paw grip force during the 1st and 2nd week of exposure to PWB. EIM values include longitudinal resistance at 50 kHz (LR 50, **A**), longitudinal reactance at 50 kHz (LX 50, **B**), and the calculated parameters of reactance-slope (X-slope, **C**), and phase-slope (P-slope, **D**). Results were analyzed with Spearman correlations and the r coefficients are displayed on the graph. *N* = 8 per group, *, **p < 0.05 and *p* < 0.01, ^δ^*p* < 0.10. A negative RPGF slope represents a decrease in RPGF. A positive weekly change in EIM represents an increase in value.

We next evaluated the relationship between the rate of change in EIM values and the RPGF occurring during both weeks 1 and 2 of unloading, since RPGF values stabilized or improved during the second week and EIM values at 2 weeks also showed opposite trends compared to their values at 7 days. Hence, we calculated RPGF slopes for the first and second week independently, a negative slope representing a decline in grip force from the previous week, and correlated these slopes with changes in EIM values during each of the same periods (Figure 4). Thus, we established correlations between RPGF slopes and weekly changes in single frequency EIM values (LR 50 kHz and LX 50 kHz, Figures 4A,B) and multi-frequency EIM parameters (X-slope and P-slope, Figures 4C,D). Our hypothesis was confirmed by the fact that LR 50, LX 50, and X-slope are inversely correlated to RPGF slopes (*r* values of -0.88, -0.95, and -0.64 with *p*-values of *p* < 0.05, *p* < 0.01, and *p* < 0.10, respectively). Interestingly, and as previously observed in this study, P-slope did not change throughout the experiment and was not correlated to changes in RPGF (*r* = -0.21).

## Discussion

In this study, we demonstrate that EIM detects muscle alterations in both micro- and partial- gravity rat analog models, and that these changes correlate with our earlier data showing alterations in RPGF in these two conditions (Mortreux et al., 2018, 2019a,c). Furthermore, we were able to confirm that the data obtained using a small intra-muscular EIM needle array yielded similar results to those previously acquired with a surface electrode (Li et al., 2013) or during *ex vivo* measurements (Sung et al., 2013) after spaceflight and HU.

It has been shown previously that muscle disuse induced by spaceflight or hindlimb unloading (HU or HLS) results in functional changes, including reductions in grip force (Hurst and Fitts, 2003; Globus and Morey-Holton, 2016; Mortreux et al., 2019c; Figure 1F). In the present study, EIM values at 50 kHz including phase and reactance parallel those observed functional changes, while resistance remains relatively stable throughout (Figures 1A–C). Fifty kilohertz is a standard frequency used in many EIM studies since muscle tends to be most reactive (i.e., exhibits the greatest capacitance) around this frequency (Mcilduff et al., 2017). However, a single frequency does not capture the full spectral character of the impedance data, and thus other measures including the phase- and reactance-slopes were introduced to try to capture those changes (Rutkove et al., 2010). As we previously observed in rats using a surface array, and in mice using *ex vivo* EIM, phase-slope decreases significantly when animals are exposed to unloading or spaceflight (Figure 1D), and correlates significantly with myofiber size (Li et al., 2013; Sung et al., 2013). In our study, this decrease in phase-slope was further associated with a significant and continuous reduction in the area under curve of the reactance multifrequency curve (AUC LX, Figure 1E). However, while the results obtained from our needle array are similar to those obtained with a surface array, it appears that measuring impedance intra-muscularly allowed us to detect changes earlier (i.e., as soon as 7 days of suspension, our earliest time-point) than when using a surface array in which EIM parameters were significantly altered later (i.e., detected after 7 days of recovery following a 14 days suspension) during exposure to HU (Li et al., 2013).

The partial weight-bearing (PWB) model also produces significant losses in muscle force and function, and result in myofiber atrophy (Ellman et al., 2013; Mortreux et al., 2018, 2019a,c). Grip force is reduced in a dose-dependent manner early on during exposure to unloading and further persists with time compared to the control animals (Figure 2F). Surprisingly, the 50 kHz EIM data did not display a significant trend over the 14-day period (Figures 2A–C). Unlike the animals exposed to HLS, phase-slopes remained unchanged during PWB while LX AUC significantly increased in the PWB20 group over time (Figures 2D,E). Therefore, we chose to evaluate EIM parameters specifically at 7 days of PWB, where the decline in grip force was sharper and dose-dependent (Figure 3A). Unexpectedly, 50 kHz values at 7 days of exposure to PWB revealed a dose-dependent relationship for all impedance measures (Figures 3B–D), in a direction opposite to that observed in HLS animals. We also detected dose-dependent changes in both AUC LX (Figure 3E) and reactance-slope (Figure 3F). The correlations with our earlier RPGF measurements across week 1 and week 2 support that these EIM changes, despite being in the opposite direction of those observed with HLS, have functional significance (Figure 4).

What factors could explain the observed impedance differences in two muscle unloading models that appear to be so closely related? First, the most obvious is that unlike HLS, animals exposed to PWB do not have a cephalic fluid shift (Globus and Morey-Holton, 1998). Stated another way, HLS animals would be anticipated to lose more water from their hind limbs than those undergoing PWB where there is no fluid shift, as the animals remain essentially horizontal throughout the 2-week suspension period. However, reductions in hind limb extracellular fluid levels would likely have the opposite effects on the EIM values observed here – i.e., we would anticipate increases in reactance/resistance in the HLS animals, rather than decreases. Moreover, the HLS animals are allowed to return to a horizontal position for a few minutes prior to EIM measurements, likely sufficient time to correct such fluid redistribution in the muscle. Second, it is possible that some other model-specific effect was occurring, such as the harness impacting venous return, but we have shown that not to be the case previously (Mortreux et al., 2020). We believe that a more likely possibility is that the HLS animals are totally non-weight-bearing on their hindlimbs and hypokinetic, whereas the PWB animals are slightly weight-bearing on their hind limbs and are able to freely move around their cages. This display of relatively normal locomotor activity could impact our EIM data in several ways, for example by increasing muscle water content (both intra- and extra- cellular) or by causing a shift from slow-twitch to fast-twitch muscle fiber types. While a decrease in reactance would be expected from a decrease in myofiber size (Kapur et al., 2018a), it is possible that the myofiber type-switch (Sanchez et al., 2014) altered the electric properties of the gastrocnemius muscle undergoing unloading. Indeed in 16 week old male rats, type 1 slow twitch and type 2 fast twitch fibers have significantly different cross section area (Type 1: 1913.8 ± 131.9, type 2: 2359.7 ± 118.2, mean difference 23.3%) (Mortreux et al., 2019a).

Finally, in addition to myofiber size, impedance measurements can be impacted by a wide range of factors including structural components (Dharia et al., 2009), interstitial fat or connective tissue deposition, and inflammation (Mortreux et al., 2019d). It is certainly possible that these two models differentially affect these muscle components. Additional histological analyses will be required to address this possibility, although our previous analysis did not highlight any inflammatory infiltration to date (Mortreux et al., 2019a,b).

Although unexpected, our results highlight a very interesting potential use of EIM: the ability to discriminate between non-weight-bearing and partial weight-bearing induced-disuse in the hindlimbs. Therefore, this technology, already available for clinical trials and human use, could be implemented during the next Artemis lunar missions, during which astronauts will be exposed to microgravity and partial gravity consecutively. Focusing on specific EIM changes characteristic of specific portions of the mission (i.e., decreased phase-slope, 50 kHz LP and 50 kHz LX for microgravity and increased reactance-slope, 50 kHz LP, 50 kHz LX, and 50 kHz LR for partial gravity), a single non-invasive EIM scanner could be used to assess muscle deconditioning and the potential impact of countermeasures. Moreover, studies have shown that electrical impedance can be used in combination with ultrasound to detect muscle alteration (Roy et al., 2019) leading to the development of new and more sensitive dual-techniques (Murphy et al., 2019). Such combinations could be very valuable for astronauts as ultrasound equipment is already available to crew members and will most likely be available for the upcoming lunar missions (Garcia et al., 2018).

While promising, our results have a number of limitations. First, we performed EIM using a needle intra-muscular array electrode designed and coated to minimize the impact of subcutaneous fat; thus, how these data relate to surface-acquired EIM data would need to be studied further. Second, unlike previous work (Mortreux et al., 2018, 2019a,b,c), the animals studied here were not euthanized at 14 days, actual tissue data was not available. Future work should focus on obtaining extensive functional and histological analysis concomitantly with EIM measurements to ascertain correlations with EIM results. Third, RPGF was not performed on the rats studied here and was instead taken from our earlier work. It is important to note in those earlier studies (Mortreux et al., 2019a) that we analyzed a very large number of animals, so the RPGF values are quite robust and have been found to be highly reproducible across our different cohorts. Finally, we only assessed muscle health and function weekly, and future Artemis astronauts will most likely spend less than a week in microgravity before landing on the Moon. Thus, it will be necessary to assess if these characteristic EIM values and parameters can also be found at earlier time-points in these ground-based models.

Taken together, our data suggest that EIM is sensitive to change in animals exposed to micro- and partial- gravity analogs, and that these models appear to impact the electrical properties of muscle differently. While future studies should seek to delve deeper into the underlying cellular mechanisms responsible for these difference, the use of a rapid and convenient technology could make it a valuable tool for astronauts to assess muscle condition en route to and during prolonged stays on the Moon or Mars.

## Data Availability Statement

All datasets generated for this study are included in the article/Supplementary Material.

## Ethics Statement

The animal study was reviewed and approved by the Beth Israel Deaconess Medical Center Institutional Animal Care and Use Committee.

## Author Contributions

CS, DR, D-MS, and MM performed the experiments. CS, D-MS, JN, and MM analyzed the results. MM prepared the figures and drafted the manuscript. SR and MM designed the experiments and conceived the research. All authors were involved in the revision of the manuscript and approved its final version.

## Conflict of Interest

SR had equity in, and serves a consultant and scientific advisor to, Myolex, Inc., a company that designs impedance devices for clinical and research use, he was also a member of the company’s Board of Directors. The company also had an option to license patented impedance technology of which SR was named as an inventor. The remaining authors declare that the research was conducted in the absence of any commercial or financial relationships that could be construed as a potential conflict of interest.

## Supplementary Material

TABLE S1 Reproducibility of the intra-muscular EIM parameters at baseline.

TABLE S2 Summary of the findings of the 2-way RM ANOVA analysis.
