# Supplementary material for: Using Electrical Impedance Myography as a Biomarker of Muscle Deconditioning in Rats Exposed to Micro- and Partial-Gravity Analogs
Source: Front Physiol. 2020 Sep 15;11:557796. doi: 10.3389/fphys.2020.557796 (PMC7522465; doi:10.3389/fphys.2020.557796)
Supplement: TABLE S1 — Reproducibility of the intra-muscular EIM parameters at baseline. [file Table_1.DOCX]

**Supplementary Tables**

**Supplementary Table 1: Reproducibility of the intra-muscular EIM parameters at baseline for the animals exposed to HLS and PWB.**

|  | **HLS (n=28)** | **PWB (n=35-59)** |
| --- | --- | --- |
| **Parameter** | **Mean±SEM** | **Mean±SEM** |
| LP 50 | 24.23±0.83 | 19.71±0.33 |
| LX 50 | 55.86±2.88 | 47.59±1.0 |
| LR 50 | 122.00+1.62 | 132.20±1.35 |
| Phase-slope | 12.60±0.97 | 11.87±0.53 |
| X-slope | - | 52.29±1.49 |

**Supplementary Table 2: Summary of the findings of the 2-way RM ANOVA analysis for figures 2-3.**

| **Parameter (Panel)** | **Time** | **PWB** | **Time x PWB** |
| --- | --- | --- | --- |
| Change in Rear Paw Grip Force (2A) | p<0.0001 | p<0.0001 | p<0.0001 |
| LP 50 (2B) | ns | ns | ns |
| LX 50 (2C) | ns | p<0.05 | ns |
| LR 50 (2D) | ns | p<0.01 | ns |
| Phase-slope (2E) | ns | ns | ns |
| AUC LX (2F) | p<0.05 | ns | p<0.001 |
| Slopes for Changes in Rear Paw Grip Force (3A) | p<0.0001 | p<0.0001 | p<0.001 |
